# Simultaneous Presbyopia and Astigmatism Correction with a Novel Trifocal Toric Intraocular Lens—A One-Year Follow-Up

**DOI:** 10.3390/jcm11144194

**Published:** 2022-07-19

**Authors:** Ladislav Viktor Nováček, Marie Němcová, Kristýna Sičová, Kateřina Tyx, Pavel Rozsíval, Jan Němčanský, Pavel Studený

**Affiliations:** 1 Department of Ophthalmology, School of Medicine Hradec Králové, Charles University Prague, 500 03 Hradec Králové, Czech Republic; pavel.rozsival@fnhk.cz; 2 Department of Ophthalmology, Institute of Aviation Medicine Prague, 160 00 Prague, Czech Republic; nemcova@ulz.cz (M.N.); sicova@ulz.cz (K.S.); tyx@ulz.cz (K.T.); 3 Department of Ophthalmology, Military University Hospital Prague, 1st Faculty of Medicine, Charles University Prague, 121 08 Prague, Czech Republic; 4 Department of Opthalmology, University Hospital Královské Vinohrady, 3rd Faculty of Medicine, Charles University Prague, 100 00 Prague, Czech Republic; pavel.studeny@fnkv.cz; 5 Department of Ophthalmology, University Hospital Ostrava, 708 00 Ostrava, Czech Republic; jan.nemcansky@fno.cz

**Keywords:** intraocular lens, astigmatism correction, presbyopia, toric, trifocal, cataract, rotational stability

## Abstract

The current investigation evaluates the efficiency of the trifocal toric Liberty 677MTY intraocular lens (IOL) in correcting preoperative corneal astigmatism in cataract patients demanding spectacle independence. The retrospective evaluation included 28 eyes of 15 patients with preoperative corneal astigmatism of at least 1.0 Dioptre (D). All patients were followed up for one year postoperatively. Residual refractive errors and visual acuities at multiple distances were measured. Binocular visual acuity and contrast sensitivity defocus curves were plotted. Visual functions and patient satisfaction were assessed. The efficiency of astigmatism correction was determined using the vector analysis method. The mean spherical equivalent refraction (SEQ) improved from 2.72 ± 1.62 D to 0.10 ± 0.48 D. The cylindric refraction decreased from 1.18 ± 0.45 D to 0.16 ± 0.31 D. Vector analysis proved efficient astigmatism correction with a centroid of 0.10 ± 0.34 D at 161°. Ninety-two percent of eyes resulted within 0.5 D from the target refraction. Visual acuities were 0.1 logMAR or better from +1.0 to −3.5 D defocus values. Visual tasks could be performed without major difficulties. Our patients were highly satisfied. Refractive and visual outcomes with the investigated presbyopia-correcting toric IOL are predictable and the lens provides excellent trifocal vision.

## 1. Introduction

Presbyopia is an age-related impairment reducing the accommodation ability of the crystalline lens, resulting in difficulties while performing near-vision related tasks such as reading, writing, or viewing digital devices [1]. The visual limitations caused by presbyopia reportedly induce distress and low self-esteem in the affected patients [1]. More than half of the middle-aged to the elderly population is affected by presbyopia, with an estimated 1.09 billion cases globally [1].

The surgical correction of presbyopia during cataract surgery or refractive lens exchange offers an efficient restoration of visual comfort at multiple distances. Several multifocal intraocular lenses (IOLs) have been introduced to the market during the last decade. However, the best possible visual outcome can only be accomplished if all refractive errors, including astigmatism, are properly corrected [2,3,4]. Ferrer-Blasco et al. reported that the majority (87%) of cataract patients had preoperative astigmatism [5], and another recent study reported that 42% of eyes had corneal astigmatism of at least 1.00 dioptres (D) [3]. This amount of astigmatism is already large enough to compromise both postoperative visual acuity and visual quality if it is left uncorrected [2,3,4,5].

Recently, trifocal toric IOLs (TTIOLs) have been developed to provide high-level visual comfort for numerous patients affected by both presbyopia and astigmatism. However, only a limited number of scientific publications are currently available on the clinical outcomes and long-term stability following the implantation of trifocal toric lenses. According to our knowledge, to date, no paper has been published on the clinical performance of the Liberty 677MTY trifocal toric lens.

Our current investigation aimed to assess the refractive and visual outcomes, including the assessment of visual quality and visual functions, and the astigmatism-correcting efficiency of the IOL named above.

## 2. Materials and Methods

### 2.1. Subjects

The current retrospective clinical investigation evaluated the pre- and postoperative records of presbyopic cataract patients, who all had preoperative corneal astigmatism. All patients expressed their demand for spectacle independence. The subjects underwent mono- or binocular cataract surgery (2 and 13 patients, respectively) performed by the same experienced surgeon (LVN) between September 2019 and December 2020. The patients included in the assessments were selected carefully. Subjects with congenital eye diseases, uveitis, amblyopia, eye trauma, previous corneal laser refractive surgery, and/or other corneal or intraocular pathologies were not included in the analyses. Only adult patients with normal anterior segment conditions (apart from cataracts), clear intraocular media and healthy retinal status were selected for evaluation.

The current clinical investigation was approved by the Ethics Committee of the University Hospital Hradec Králové (Reference number: 202204 P01; 8 March 2022). The principles of the Declaration of Helsinki were followed during the data management and the entire clinical evaluation [6].

### 2.2. Pre- and Postoperative Examinations

Before surgery, each patient underwent detailed ocular examination including corneal topography, aberrometry (TRK-2P, Topcon Corporation, Tokyo, Japan), anterior segment examination (Oculus Pentacam 70700, OCULUS Optikgeräte GmbH, Wetzlar, Germany), posterior segment examination with a slit lamp (SL-D4, Topcon, Topcon Corporation, Tokyo, Japan), and optical biometry measuring axial length (AXL), anterior chamber depth (ACD), and keratometry values (K1, K2; IOL Master 700, Carl Zeiss Meditec AG, Jena, Germany). Intraocular pressure was recorded in all cases.

Cataracts were diagnosed based on slit-lamp evaluation and the subjective loss of vision in the case of each subject.

Monocular uncorrected and corrected visual acuities (distance UDVA, CDVA at 4 m; intermediate UIVA, CIVA at 67 cm; near UNVA, CNVA at 35 cm) were assessed using the Vision C iPad application (Clínicas Qvision, Almería, Spain) [7].

The same measurements were performed one day, one week, one, three, and six months, and then one year postoperatively.

Additionally, binocular visual acuity and contrast sensitivity defocus curves were plotted in photopic conditions using the Multifocal Lens Analyzer 3.0 (MLA) iPad application by Clínicas Qvision, Almería, Spain [8]. The recommended protocol of the developers was applied in all cases.

Contrast sensitivity measurements were performed using the CSV-1000 device (VectorVision, Houston, TX, USA) under photopic, photopic with glare and mesopic light conditions.

Our retrospective evaluation included a vector analysis with the Alpins method, which provides additional information on the astigmatism-correcting ability of the IOL in question.

Rotational stability was evaluated by comparing the position of the IOL during each postoperative visit to the surgical position. Rotation was expressed as the mean absolute rotation (focusing on the degree of rotation), as well as the signed rotation, which also takes the direction of the rotation into account. Clockwise rotation was counted as negative and counterclockwise as positive rotation. Rotation between the intended torus position and that measured on the first postoperative day was regarded as misalignment, while later on as toric IOL rotation.

Visual functions, quality of vision, and patient satisfaction were evaluated with a standard questionnaire one, three, and six months and then one year following surgery.

### 2.3. The Bi-Flex Liberty 677MTY Intraocular Lens

The Liberty 677MTY trifocal toric IOL (Medicontur Medical Engineering Ltd., Zsámbék, Hungary) is a hydrophilic, acrylic, single-piece capsular bag lens designed for presbyopia correction. The optic uses the diffractive principles with seven diffractive rings in the centre of the optics while leaving the outer 75% of the lens surface purely refractive. The two toric marks indicate the flat axis on the edge of the optic. The optic is connected to two double-loop haptics without haptic angulation. The IOL has a 360° square edge (also in the optic-haptic junction) to prevent the development of posterior capsule opacification (PCO). The IOL material has 25% water content and includes a UV-absorber and a blue-light filter.

The online calculator of the manufacturer [9] was used for IOL calculation in each case. The target refraction was Plano (emmetropia) in each case, and the personal surgically-induced astigmatism (0.25 D) of the surgeon was always taken into account. The optimal spherical equivalent (SEQ) and cylindrical power (CYL) of the IOL were determined by the Haigis formula [10] for each eye. Posterior corneal astigmatism was considered during the optical biometry measurements performed by the IOL Master 700.

### 2.4. Surgical Technique

All surgeries were performed under local anaesthesia and following pupil dilation according to the standard cataract surgery protocol using the phacoemulsification method described previously [11]. Clear corneal incisions of 1.7 mm with an extension to 2.2 mm were made at 135°. The toric IOL was positioned by accurately aligning the toric reference marking of the IOL using the toric IOL alignment tool of the Callisto eye system (Carl Zeiss Meditec AG, Jena, Germany).

### 2.5. Vector Analysis

The cumulative histogram of the magnitudes of the preoperative corneal astigmatism and postoperative refractive astigmatism at the corneal plane (measured 12 months postoperatively) and the double-angle plots of preoperative and postoperative refractive astigmatism (including centroid values with standard deviations and 95% confidence ellipses of the dataset and the centroid values) were plotted according to the method presented by Abulafia et al. [12].

### 2.6. Statistical Analysis

All data were recorded in Microsoft Excel. Evaluations were performed using the GraphPad Prism 8.3.0 software (GraphPad Software, San Diego, CA, USA). After de-identification, the complete dataset evaluated in the current work were made available in the Mendeley Data depository database (https://doi.org/10.17632/58xgbgsx8b.1, accessed on 14 April 2022) [13].

Mean; standard deviation, SD; median; minimum; maximum; and 95% confidence intervals were calculated for all variables. Each parameter was tested for normal distribution using the D’Agostino and Pearson test. Comparisons between matching pre- and postoperative variables were performed using either the paired two-tailed *t*-test or the Mann–Whitney test, as required. A statistical significance level of *p* ≤ 0.05 was applied in each case.

Clinical outcomes are presented according to the standards for reporting refractive outcomes of intraocular lens-based refractive surgery [14].

## 3. Results

The pre- and postoperative records of 28 eyes of 15 patients (9 females and 6 males) were collected. Table 1 introduces the demographics and baseline optical values of the study population. The average age of the cohort was 52.4 ± 5.26 years. One patient was not eligible for the six-month visit, while another one could not attend the one-year check-up.

IOL-related complications could not be observed during surgery. Intraocular pressure was maintained in each case (<20 mmHg) during and after the operation (data not shown). One eye showed symptoms of anterior uveitis one month postoperatively, while two eyes developed endothelopathy three and six months following surgery. These conditions had cleared up within one month in all affected cases. No further adverse events that may be correlated with either the surgery or the IOL implantation occurred during the first postoperative year; however, one patient was diagnosed with dry-eye syndrome one year after surgery.

### 3.1. Refractive Outcomes–Astigmatism—Correction

**Figure 1:**
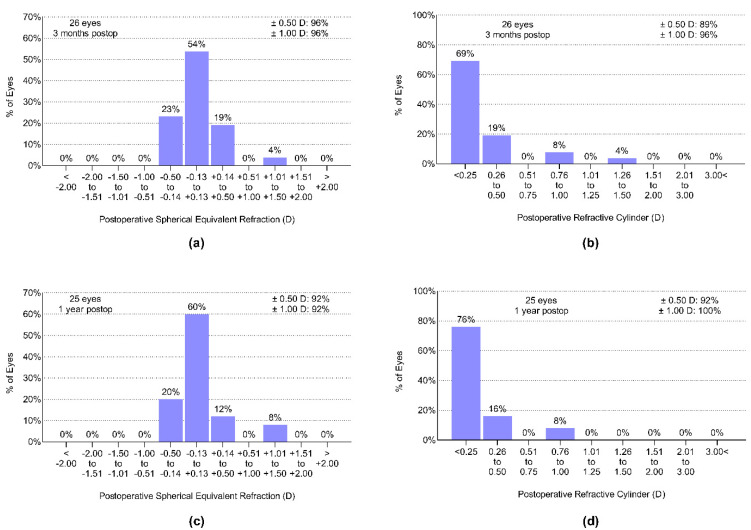
Spherical equivalent refraction and residual refractive cylindric refraction measured three months (**a**,**b**) and one year (**c**,**d**) postoperatively.

The average preoperative spherical equivalent refraction of the patient group was 2.72 ± 1.62 D. Three months postoperatively, 96% of eyes were within 0.5 D, and 96% were within 1.0 D (Figure 1a) from the target refraction, emmetropia. The average subjective refractive astigmatism was −0.41 ± 0.46 D before surgery. The cylindrical correction was found to be effective: 89% of eyes had a residual astigmatism of not more than 0.5 D, and 96% of the eyes were within 1.0 from Plano (Figure 1b). The refractive outcomes remained stable during the first postoperative year in the majority of the cases (Figure 1c,d).

### 3.2. Vector Analysis

**Figure 2:**
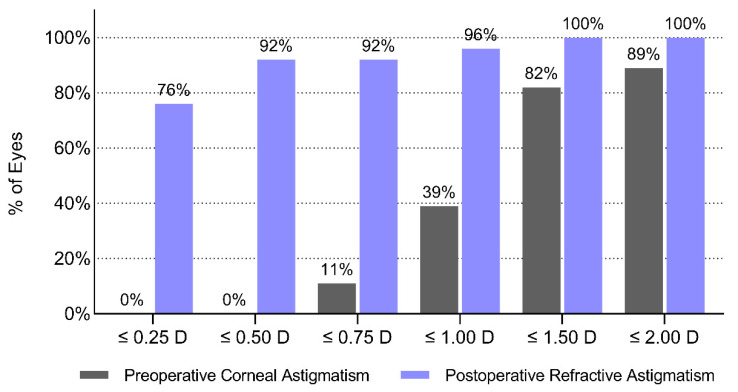
Cumulative histogram of preoperative corneal and postoperative refractive astigmatism measured one year postoperatively at the corneal plane.

**Figure 3:**
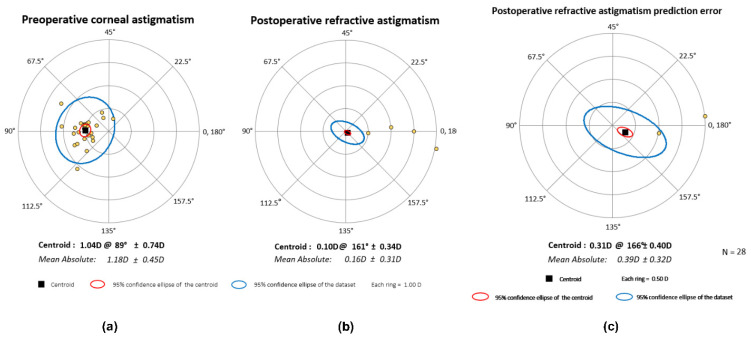
Double-angle plots of preoperative (**a**) and refractive astigmatism (**b**) and of the prediction error (**c**) measured and calculated one year after surgery.

The astigmatism-correcting capacity of the study lens was examined with vector analysis. The magnitudes of the preoperative corneal and postoperative refractive astigmatism (measured 12 months after surgery at the corneal plane) are shown in the cumulative histogram, Figure 2. Figure 3 shows the double-angle plots of preoperative and postoperative refractive astigmatism. Centroid values and the 95% confidence ellipses are indicated in each graph. While the preoperative mean absolute corneal astigmatism was 1.18 ± 0.45 D, a mean residual refractive astigmatism of 0.16 ± 0.31 D could be measured twelve months postoperatively.

### 3.3. Rotational Stability of the IOL

The rotational stability was determined by comparing the position of the toric marks of the IOL at each postoperative visit to the surgical position of the lens. There was no need for repositioning in any of the cases. Table 2 presents the average signed and absolute rotations during each postoperative visit. While the signed rotation takes the direction of the rotation into account, the absolute rotation disregards the direction and only considers the magnitude of rotation.

### 3.4. Visual Outcomes

A remarkable gain could be achieved in monocular uncorrected and corrected visual acuities at multiple distances, compared to the preoperative values (Table 3). UDVA increased from 0.54 ± 0.30 logMAR to 0.02 ± 0.08, and UNVA improved from 0.31 ± 0.42 to 0.19 ± 0.12 three months postoperatively, and to 0.21 ± 0.13 one year postoperatively. All measured values remained practically unchanged thereafter; however, a significant improvement in binocular distance-corrected near vision (DCNVA) could be observed between the third and twelfth postoperative months (*p* = 0.0176).

**Figure 4:**
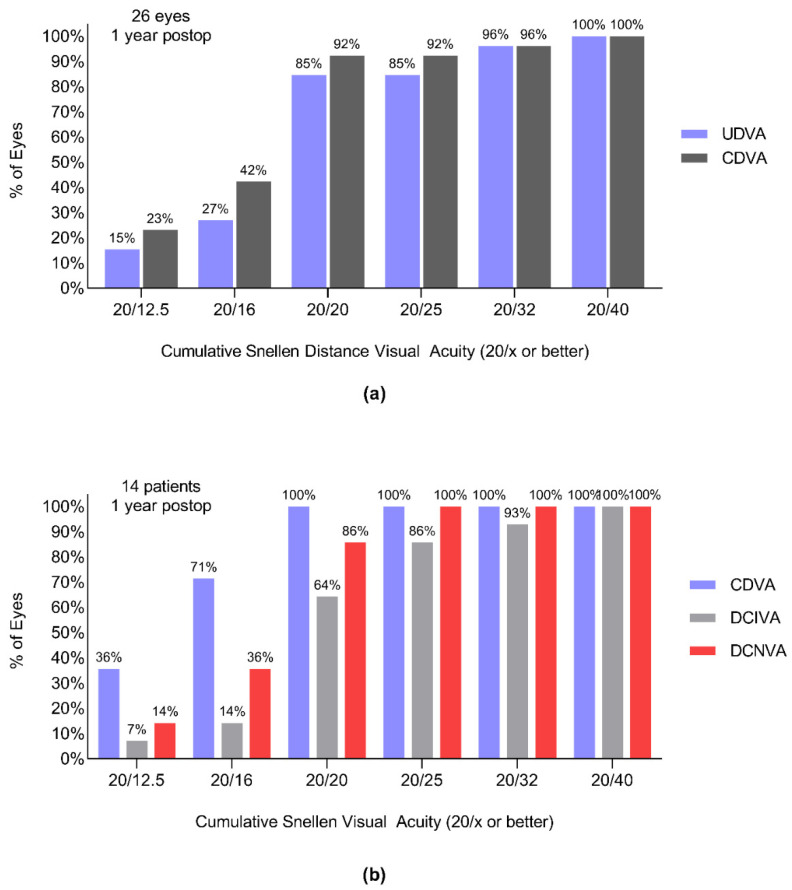
(**a**) Cumulative histogram of monocular uncorrected and corrected distance visual acuity. (**b**) Cumulative histogram of binocular distance-corrected visual acuities at multiple distances measured 1 year postoperatively.

Figure 4a demonstrates the cumulative percentage of eyes in each category of monocular distance visual acuity. The monocular UDVA of 80.8% of the eyes was the same or better than the CDVA, while 92.3% of eyes were within one line of CDVA. The binocular distance-corrected intermediate and near visual acuities are shown in Figure 4b.

**Figure 5:**
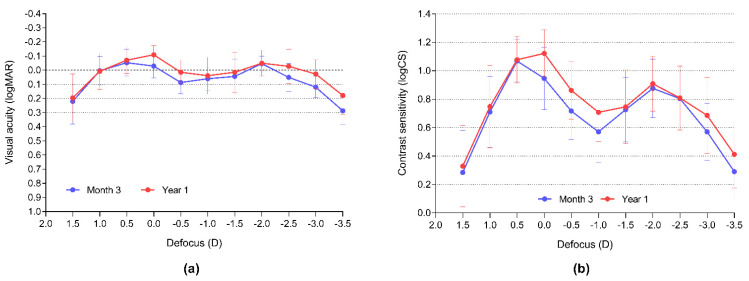
Binocular visual acuity (**a**) and contrast sensitivity (**b**) defocus curves confirm stable visual outcomes.

Binocular visual acuity defocus curves were maintained during the one-year follow-up (Figure 5a). During the last visit, the mean VAs were 0.1 logMAR or better from +1.75 D to −3.25 D defocus (5.0 D wide defocus range). Contrast sensitivity defocus curves, which are reported to be more precise in reflecting small changes in optical quality than VA [15,16], show a non-significant improvement in the far and intermediate ranges between the third and twelfth postoperative months (Figure 5b).

### 3.5. Visual Quality

**Figure 6:**
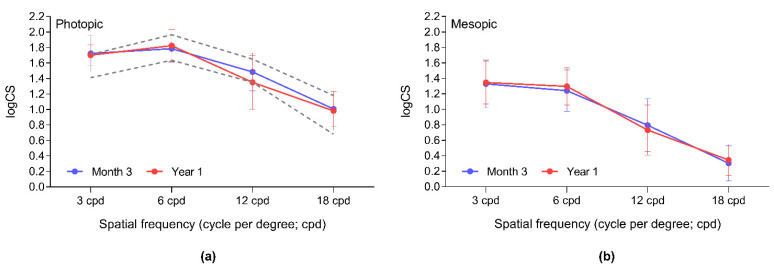
Photopic (**a**) and mesopic (**b**) contrast sensitivity is stable during the first postoperative year. Results are within the age-defined population normal values for the older age group (50–75 years of age) [17]. logCS: logarithm of Contrast Sensitivity; cpd: Cycle per degree.

Contrast sensitivities measured under different light conditions were stable post-surgery; no significant change could be revealed between the 3-month and 1-year results (data not shown; Figure 6).

**Figure 7:**
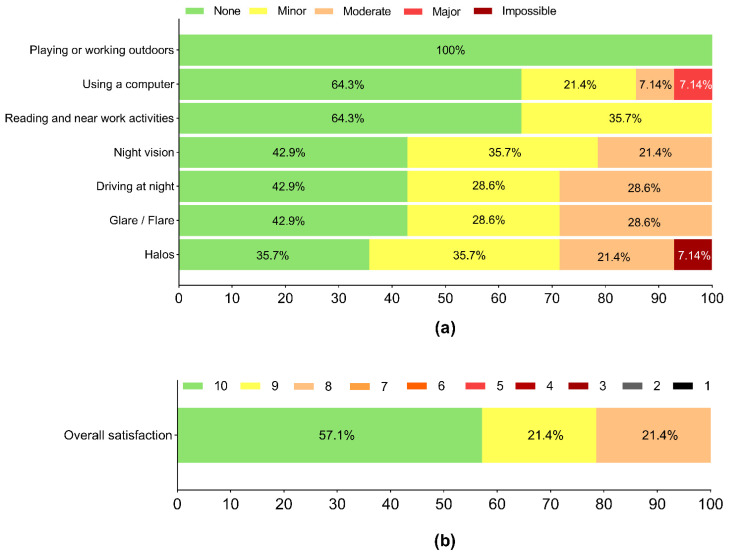
Visual function questionnaires reflected no or minor difficulties while performing general daily activities (**a**,**b**), and high patient satisfaction on a scale of 1 to 10 (1 = lowest satisfaction, 10 = highest satisfaction). Horizontal axes represent the percent (%) of patient responses, while colours represent the possible responses.

The majority of patients had no, or only mild, difficulties performing their usual daily activities (Figure 7a), and all of them were highly satisfied with their vision (mean rating of 9.14 ± 1.23 on a scale from 1 to 10, where 10 represents the highest level of satisfaction; Figure 7b).

## 4. Discussion

Our current work aimed to evaluate the refractive and visual outcomes, including the assessment of visual quality and visual functions, and the astigmatism-correcting efficiency of the astigmatism correction Bi-Flex Liberty 677MTY trifocal toric capsular bag IOL.

The pre- and postoperative data of 15 cataract patients recorded during the first year following surgery were collected and evaluated. Implantation and the investigated IOL were shown to be safe, as no persistent complication directly related to the intervention could be registered.

Astigmatism correction was found to be efficient: the majority of eyes achieved postoperative refraction close to or at the target refraction, emmetropia. Our figures are similar to those we reported earlier following the implantation of the Bi-Flex 677TAY monofocal toric IOL [11] and are somewhat better than those reported by Bachernegg et al. for the same monofocal toric lens [18]. As expected, the current residual SEQ is lower than that observed after the implantation of the Bi-Flex Liberty 677MY trifocal non-toric IOL model [19], as additional astigmatism correction improved the final refractive outcomes. The predictability of the results is found to be higher than that reported for other TTIOLs such as the FineVision Pod FT [20], and AT LISA tri 939MP [21,22]. In contrast to the findings of Vanderkerckhove et al. [23], who found that 81.1% of the eyes were within 0.5 D from the target refraction 6 months following the implantation of the FineVision PodFT IOL, but only 65% 1 year postoperatively, our results remained unchanged during the one-year follow-up (89% and 92%, respectively), suggesting refractive stability. Vector analysis confirmed the efficiency of astigmatism correction, and our findings are in good agreement with those of the same but monofocal IOL model [11].

Rotational stability was high and similar to that reported on the monofocal toric model of the same lens [11,18]. Mean IOL-rotations were comparable with those published on TTIOLs with modified L- or double C-loop haptic designs [24]; however, unlike with AT LISA tri 939MP and FineVision PodFT, no IOL-repositioning was required [19,20]. A stable IOL position contributed to the maintained refractive and visual outcomes. Our findings are in agreement with the previous reports [25,26], claiming that toric IOL rotations of less than 10 degrees do not change the eye’s refraction by less than 0.50 D.

Visual outcomes reflect the refractive results. Visual acuities and defocus curves confirm efficient presbyopia correction. The mean binocular distance-corrected visual acuities were 0.1 logMAR or better from +1.25 to −3.25 D defocus values, which are equal to infinite to cc. 30 cm reading distance. This range is wider than that reported for the PanOptix Toric and FineVision Pod FT IOLs (3.75 D range) [24,27], or for the AT LISA Tri Toric model (3.00 D range) [28].

Photopic contrast sensitivities were within the normal range for the elderly population [29]. Mesopic CS values were close to the normal values for lower spatial frequencies except for 18.0 cpd, which was within the normal range, similar to that reported on a plate-haptic TTIOL [27]. Both our photopic and mesopic measures are comparable with those reported on the non-toric Bi-Flex 677TAY model [19,30]. Although CS measurements are difficult to compare due to their subjective nature and various scales used in reporting the results, we could not detect remarkably lower CS values at higher spatial frequencies as was seen with other TTIOLs [22,24,31].

Similar to previous findings with other TTIOLs in the literature [27,28], our patients were able to perform their daily visual tasks without any, or only minor difficulties. High satisfaction was reported in each case. The limitation of our investigation is that we did not perform a detailed dysphotopsia evaluation; however, none of our patients complained about intolerable photic disturbances. Previous works with the non-toric trifocal 677MY IOL reported rare and minor occurrences of dysphotopic phenomena [19,30,32,33].

Our work has several limitations. We evaluated patient records collected retrospectively, and due to the COVID-19 pandemic, several patients could not attend all pre-scheduled visits. Hence, only a limited number of patients could be enrolled in the analysis. Visual functions were tested with our regularly used, but non-validated, questionnaire; therefore, the comparison of the results with other results published in the scientific literature is difficult.

The main advantage of our investigation might be the relatively long (one-year) follow-up period, which is rare among the scientific papers published on TTIOLs. Only four publications with similar longitudinal monitoring could be found in the literature [21,23,31,34]. Moreover, according to our knowledge, this is the first paper published on the refractive and visual outcomes following the implantation of the Bi-Flex 677MTY lens.

## 5. Conclusions

Our findings suggest that the TTIOL in focus is safe and efficient in the simultaneous correction of presbyopia and preoperative corneal astigmatism. The patients’ vision is restored in a wide defocus range, and good visual quality enables them to perform various tasks independently of the required distance. The IOL position and hence the refractive and visual outcomes are found to be stable, which is the key to long-term patient satisfaction. Further examinations and a larger number of subjects are required to confirm the present findings, but our results suggest that the examined TTIOL is a good option for astigmatic patients with high visual expectations.

## Figures and Tables

**Table 1:** Demographic and baseline characteristics of the subjects.

Demographic	Mean ± SD	Range
Age (y)	52.4 ± 5.26	45–64
Sex (n)		
Female	9 (60.0%)	
Male	6 (40.0%)	
AXL (mm)	22.66 ± 1.00	20.80–24.72
ACD (mm)	3.04 ± 0.29	2.54–3.02
K1 (mm)	7.91 ± 0.27	6.98–8.27
K2 (mm)	7.69 ± 0.23	6.89–8.05
CYL (D)	−0.45 ± 0.69	−2.75–−0.50
SEQ (D)	2.78 ± 1.67	0.00–6.75
UDVA (logMAR)	0.54 ± 0.30	1.10–0.30
CDVA (logMAR)	0.01 ± 0.07	0.20–−0.10
UNVA (logMAR)	0.31 ± 0.42	0.90–0.20
CNVA (logMAR)	0.17 ± 0.11	0.20–0.00
IOP (mmHg)	15.1 ± 2.86	11.0–20.0
IOL power (D)	24.6 ± 3.69	17.5–33.0
IOL Cylinder (D)	1.23 ± 0.40	1.00–2.50

AXL: Axial length; ACD: Anterior chamber depth; K1 and K2: Keratometry values; CYL: Cylindric refraction; SEQ: Spherical equivalent refraction; D: Dioptre(s); UDVA: Uncorrected Distance Visual Acuity; CDVA: Corrected Distance Visual Acuity; UNVA: Uncorrected Near Visual Acuity; CNVA: Corrected Near Visual Acuity; IOP: Intraocular Pressure; IOL: Intraocular lens.

**Table 2:** Average absolute and signed off-axis rotation 3 months and 1 year following IOL implantation ^1^.

Follow-Up Visit	Signed	Absolute
Month 3	−0.68 ± 2.58	2.04 ± 1.67
Month 6	0.04 ± 3.03	2.27 ± 1.95
Year 1	−0.35 ± 3.86	2.96 ± 2.42

^1^ Results are presented as mean ± SD and expressed in degrees (°).

**Table 3:** Pre- and postoperative visual acuities (distance, intermediate, near) ^1^.

Visual Acuity (logMAR)	Preop	Month 3	Year 1	Preop vs. Month 3 *p* =	Month 3 vs. Year 1 *p* =
**Monocular**					
UDVA	0.54 ± 0.30	0.02 ± 0.08	−0.01 ± 0.12	<0.0001	0.0861
CDVA	0.01 ± 0.07	−0.02 ± 0.05	−0.05 ± 0.12	0.0898	0.4126
UIVA	n.m.	0.18 ± 0.20	0.19 ± 0.16	n.a.	0.8438
CIVA	n.m.	0.02 ± 0.09	−0.01 ± 0.11	n.a.	>0.9999
UNVA	0.31 ± 0.42	0.19 ± 0.12	0.21 ± 0.13	0.0007	0.7051
CNVA	0.17 ± 0.11	0.15 ± 0.08	0.17 ± 0.10	0.4442	0.6133
**Binocular**					
CDVA	n.m.	−0.05 ± 0.11	−0.06 ± 0.09	n.a.	0.3125
DCIVA	n.m.	0.05 ± 0.08	0.04 ± 0.13	n.a.	0.3613
DCNVA	n.m.	0.11 ± 0.09	0.03 ± 0.11	n.a.	0.0176

^1^ Results are presented as mean ± SD. n.m. = not measured; n.a. = not applicable. *p* values are the significance values from the Wilcoxon matched-pairs signed-rank tests. *p* values of not more than 0.05 were considered statistically significant. UDVA: Uncorrected Distance Visual Acuity; CDVA: Corrected Distance Visual Acuity; UIVA: Uncorrected Intermediate Visual Acuity; CIVA: Corrected Intermediate Visual Acuity; UNVA: Uncorrected Near Visual Acuity; CNVA: Corrected Near Visual Acuity; DCIVA: Distance-corrected Intermediate Visual Acuity; DCNVA: Distance-corrected Near Visual Acuity.

## Data Availability

All pre- and postoperative data collected during the study are available after de-identification in the Mendeley Data depository database from https://doi.org/10.17632/58xgbgsx8b.1 (accessed on 14 April 2022) [13].
